# Supplementary material for: Oral Administration of Lactobacillus sakei CVL-001 Improves Recovery from Dextran Sulfate Sodium-Induced Colitis in Mice by Microbiota Modulation
Source: Microorganisms. 2023 May 22;11(5):1359. doi: 10.3390/microorganisms11051359 (PMC10222668; doi:10.3390/microorganisms11051359)
Supplement: Supplementary file 1 [file microorganisms-11-01359-s001.zip › microorganisms-2391551-supplementary.pdf]

Supplementary Figure S1. Evaluation of mRNA expression of cytokine and genes coding for intracellular junction with DSS-induced colitis under co-housed condition

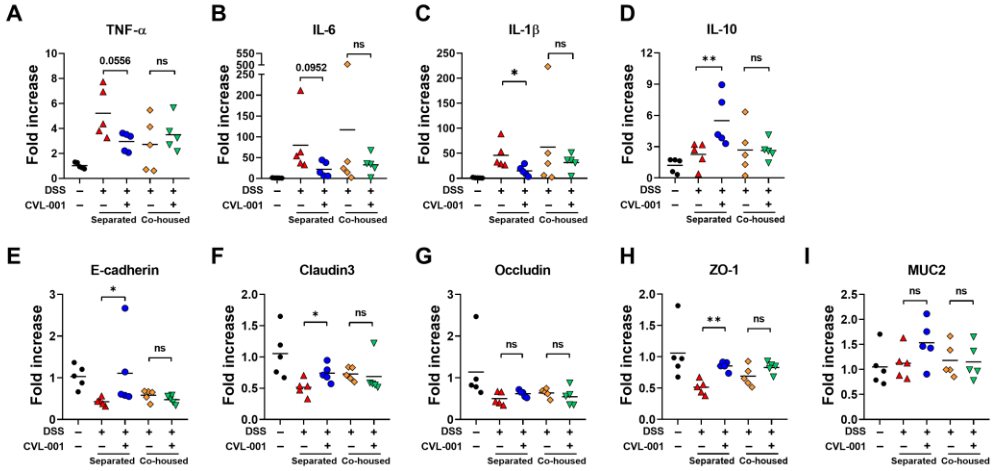

Figure S1. mRNA extracted from colonic tissue prepared for real-time PCR. The mRNA levels of **(A)** *TNF-α*, **(B)** *IL-6*, **(C)** *IL-1β*, **(D)** *IL-10* and genes coding for **(E)** E-cadherin, **(F)** claudin3, **(G)** occludin, **(H)** ZO-1, and **(I)** MUC2 were evaluated using each primer and normalized against GAPDH (n = 5). The significance of differences between the group was assessed using Mann-Whitney U-test, with the level of significance set at P < 0.05 (*). PCR, polymerase chain reaction; TNF, tumor necrosis factor; IL, interleukin; ZO, zonula occludens; MUC, mucin; GAPDH, glyceraldehyde 3-phosphate dehydrogenase; DSS, dextran sulfate sodium.
